# Physiologie Végétale

**Published:** 1834-10-01

**Authors:** 


### Physiologie Vegetale. Par *M. A. P. De Caudolle.* Tome 3. Paris. Béchet Jeune, 1832.

These three volumes are devoted to the physiology of vegetables, being a continuation of a work which is to embrace the whole science of plants. In 1827 M. De Caudolle published two volumes on the structure of vegetables; he has now added three others on their vital functions, and if time, health, and inclination permit, he promises to complete the series by discussing the principles of classification, geographical, and fossil botany, and the application of the whole science to the cultivation of plants, and to their dietetic and medicinal uses. He tells us that he has revised this work completely at four different periods; in 1804, when he delivered his first course at the College de France; in 1812, when lecturing at Montpellier; in 1829, when preparing a course of Botany at Geneva; and when bringing out the present volumes. The Royal Society, which numbers among its members many botanists most capable of estimating the value of this undertaking, have very lately shewn their high sense of the estimation in which they hold it, by presenting to M. De Caudolle, their gold medal. The botanical student who has had access to adequate libraries, and has experienced the labour which the perusal of many monographs on the same subjects by different writers demands, the confusion produced by opposite statements, and the difficulty of forming any correct judgment on disputed points, will hail with pleasure a comprehensive summary of the actual state of the knowledge of this important branch of physiology, from the pen of one eminently qualified by his learning, opportunities, and industry, to collect facts and experiments relating to vegetable life, and by his practical knowledge of the subject and mature judgment to decide on their real value. For as Montaigne has wisely said, "ce n'est pas assez de compter les expériences; il les fault poiser et assortir, et les fault avoir digérés et alambiguées pour en tirer les raisons et conclusions qu'elles portent."

The arrangement of these volumes is clear and methodical, each section corresponding with another section in the work which preceded it, so as to afford every facility of reference to the description of the part whose vital functions are explained. There are five principal sections; the first is devoted to the general phenomena of vegetable life; the second to vegetable nutrition, including the ascent and descent of the sap, the chemical changes which the atmospheric air undergoes, the various secretions and excretions, with a detailed chemical history of the numerous vegetable principles; the third section explains the reproduction of plants, a subject embracing their generation, gestation, and germination, their multiplication by division; hybrids, varieties, monsters, &c.: the fourth contains an account of certain phenomena common to nutrition and generation; such as abortion, metamorphosis, grafting, the direction of plants or of parts of plants; their movements, temperature, odour, colour, taste, consistence, age, the real or apparent suspension of vitality, transplantation, and temperament: the fifth section comprehends the action of external agents on plants; such as light, electricity, temperature, air, water, and soils, injuries accidental or otherwise, the effects of poisons, the ravages of animals, the influence of parasitical plants on others, and of the proximity of plants of the same species. There are few points in vegetable physiology of more general interest than the changes produced in the atmospheric air, by vegetables, and as M. De Caudolle has given the most lucid explanation of the phenomena that we remember to have read, we will give a condensed abstract of the chapter. Bonnet having placed some green leaves under water where they were exposed to the sun, saw bubbles of air rise from their surfaces. In order to determine whether this air came from the leaf, or the water, he placed the same leaves in the same circumstances under water that had been deprived of air by ebullition, and as no bubbles of air were produced he concluded that the phenomenon did not depend on any action of the leaf itself. This apparently logical experiment, led him however to overlook one of the most important facts in vegetation. Thirty years afterwards, Priestly, when engaged in his experiments on gases, observed the same phenomenon. He however collected the bubbles of air, and by analysis discovered that they were nearly pure oxygen gas. This attracted the attention of vegetable physiologists, particularly of Senebier and T. de Saussure, who proved it to be connected with the most important laws of vegetable life. The conditions indispensable to the production of this gas are, the green colour of the plant, the direct rays of the sun, and the presence of carbonic acid gas in the water. Experiment shews that no parts which are not green, as roots, old trunks, petals, stamens, &c. with but one or two exceptions of coloured leaves, will produce oxygen gas. The green part of the plant must also be alive, for dead plants, although still green, evolve no oxygen. This evolution does not take place unless the immersed leaves are exposed to the direct action of the sun; the clearest diffused light, or the brightest lamps produce no such effect. Finally, all kinds of water are not suitable; the action does not go on if the water has been deprived of air by distillation or by boiling, or if it has been impregnated with nitrogen, hydrogen, or oxygen gases; but, on the contrary, if the water contains carbonic acid gas, then the green leaf, aided by the action of the direct solar rays disengages oxygen. The following experiment of Senebier's, which is one among many hundreds, is conclusive. A branch of a raspberry bush which produced no gas in distilled water, furnished in common water a volume of gas equal to 108 grains of water; and in water charged artificially with carbonic acid gas, a volume of gas equal to 1664 grains of water. From such facts he concluded that the carbonic acid gas dissolved in the water is under the influence of the direct solar rays decomposed by the green parts of vegetables, the leaf most probably fixing the carbon, whilst the oxygen, becoming free, escapes. The same physiologist proved by the following experiment, that leaves also decomposed the carbonic acid which was transmitted to them by the roots, that is, dissolved in the fluid which they absorb from the soil. He placed two branches of a peach tree beneath receivers containing water, the lower ends of the stems which protruded were placed in bottles, one of which contained water charged with carbonic acid gas, the other was empty. The branch whose extremity was plunged in water charged with carbonic acid gas disengaged a quantity of oxygen gas equal to 4815 grains of water, whilst the oxygen gas disengaged by the other was only equal to 2535 grains of water. Thus, nearly half of the oxygen gas exhaled by the first appeared to be furnished by the water in contact with the leaves, and the other half by that of the water absorbed by the extremity of the stem. This explains why some fleshy leaves give out oxygen under distilled water, as their sap contains a little carbonic acid gas. If deprived of the air they contain by being placed beneath the receiver of an air-pump they then give out no oxygen gas when immersed in distilled water. That the carbon remains fixed in the plant appears from several reasons very probable. 1. This is the only means in the present state of our knowledge for accounting for the deposition of carbon in vegetables. 2. The green matter of leaves contains a great proportion of carbon. 3. Plants deprived of solar light, or etiolated, do not disengage oxygen gas, and contain less carbon than others. Some experiments of M. Goeppert render it probable that the quantity of carbonic acid never augments in these plants during vegetation, so that which was originally contained in the seed from which each sprang, becomes spread through the whole tissue of the plant. As in these experiments the plants have been immersed in water, and therefore placed in an unnatural situation, some physiologists have doubted their conclusiveness under other circumstances, particularly as no difference can be detected in the composition of the air in forests or in deserts. However, the mobility of the air would account for this, and direct experiments have been made to resolve the question. Thus, Palmer placed green branches in a vessel containing atmospheric air exposed to the sun's rays, and after 10 or 12 hours found an increase of about a fiftieth part of oxygen in the vessel. M. Theodore de Saussure has endeavoured to accomplish the same end by placing vegetables in circumstances more similar to their natural condition. Thus he has grown plants both in the sun and in the shade in an atmosphere containing small and ascertained quantities of carbonic acid, and he has found that in the sun plants flourished in an atmosphere which contained not more than a twelfth of carbonic acid gas, whilst in the shade the smallest additional quantity of this gas was injurious to the vegetation. His most instructive experiment however was the following. He reared many periwinkles from seeds, and by a previous analysis ascertained the average quantity of carbon contained in the young plants of a known weight and size. He placed seven in a receiver containing $7\frac{1}{2}$ per cent. of carbonic acid gas mixed with atmospheric air, their roots being plunged in distilled water, and seven others in similar circumstances with the exception of the air being deprived of all carbonic acid gas. He exposed the receivers to the sun. Six days afterwards he removed the plants, which were uninjured; there was no carbonic acid gas in the first receiver, but $24\frac{1}{2}$ per cent. of oxygen instead of 21. The carbonic acid had thus been decomposed by the periwinkles, but the whole of its oxygen had not been exhaled; the periwinkles themselves were found on analysis to contain 2,28 grains of carbon more than before the experiment, whilst those which had lived in the air deprived of carbonic acid gas had lost a little carbon. Similar experiments on four other plants gave the same result, with some variation in the quantity. The experiments prove that the green parts of plants in the sun decompose the carbonic acid of the air, appropriate its carbon, which increases their solid matter, retain a small quantity of oxygen, and set the rest free. In total darkness this change does not go on, but it is far from improbable that in the diffused day-light the decomposition may take place, although too feebly to be appreciated by analysis. For, 1st, if, as every thing leads us to believe, the green colour is owing to the decomposition of carbonic acid gas, the pale but decidedly green hue of those plants exposed to diffused day-light alone, proves that the same action must be going on, although more feebly. M. De Caudolle has also seen etiolated plants become green when exposed to the light of six lamps, without their disengaging a *sensible* quantity of oxygen gas. 2. As plants die when deprived of every means of decomposing the carbonic acid gas of the air, and yet live and often prosper in the shade, it is probable that they must even under such circumstances decompose a small quantity.

De Saussure has discovered that if green leaves are placed in a receiver of atmospheric air during the night the quantity of nitrogen is not sensibly altered, but that some oxygen has disappeared. Fleshy and marsh plants inspire the least oxygen, trees in general more than herbs, and trees with deciduous leaves more than those which are persistent, and young leaves of all sorts more than old ones. This oxygen inspired by the green parts of vegetables during the night does not remain in an elastic state, for neither the air-pump nor heat can disengage it. It does not become incorporated with the solid part of the plant, because the action of solar light disengages it easily. It appears, then, that at the time of inspiration it is united with the carbon dissolved in the sap, and that it forms there carbonic acid gas, during the night, which is anew decomposed by solar light. The plant then appropriates the carbon, and a small quantity of oxygen, whilst the rest of this gas, mixed with a little azote existing in the tissues, is exhaled in the air during the day.

The relation which those parts of a plant which are not of a green colour hold to the atmospheric air may be reduced to this simple law, that all these organs do not assimilate the oxygen of the air, but both by day and night this oxygen abstracts a portion of their carbon, and thus forms a certain quantity of carbonic acid gas, which sometimes becomes free in the atmosphere, at other times is dissolved, either in surrounding water, or in the water of vegetation, and thus in both cases may be re-decomposed by the green parts. This decarbonization of those parts of plants which are not green appears to be necessary to their health. Trees suffer if their roots are buried so deeply as to prevent the access of the atmospheric air to them. It is partly owing to the same cause that trees whose roots are inundated suffer and die; that light soils are more suitable to vegetables having long roots; that at a certain depth the radicles are protruded horizontally, and not vertically; that the lateral roots are in general nearer the surface of the soil; that roots suffer more from the contact of stagnant water, although richer in nutritive matters than from running water, which brings to them incessantly a little oxygen, and that finally, those roots which live in water-pipes, where there is little oxygen, appear to be obliged to multiply their surface, by pushing out a multitude of little roots in order to abstract a greater quantity of the gas. De Saussure has confirmed these practical results by direct experiments. He placed the roots of young chesnut trees in contact with different gases, and found that those whose roots were plunged in gases deprived of free oxygen, died in a few days, whilst those placed in atmospheric air prospered. The latter diminished the quantity of oxygen gas and formed with it by means of their own carbon a corresponding quantity of carbonic acid gas. Bulbs, rhizomas, and in general the subterranean parts of stems which are not green are in the same condition as roots. Flowers not only part with a portion of their carbon to the oxygen of the air, but exhale a little nitrogen ; the quantity varying from 1,500 to 45,500 of their volume. It is well known that carbonic acid gas is formed during germination, and that this decarbonization is necessary to their growth. This action of the air on those parts of vegetables which are not green cannot be considered as a truly vital effect, but as a chemical property inherent in these bodies. Indeed, this action goes on in wood and bark after death ; and Rumford has proved by direct experiments, that carbon, which has been considered as one of the most fixed bodies that is known, may become united with oxygen and form carbonic acid gas at a temperature much below that at which carbon will visibly burn. The order of arrangement of this series of composition and decomposition of carbonic acid gas is somewhat obscure. M. De Caudolle proposes the following arrangement as the most natural.

1. The water entering vegetables through their roots is charged with carbonic acid, which is carried along with the sap to the green parts where it is decomposed by the solar light ; the carbon is fixed, and the oxygen escapes in the form of gas.

2. The carbonic acid gas which those parts of vegetables which are not green have formed with the oxygen of the air, is partly dispersed in the atmosphere, partly dissolved in the water of vegetation, and carried with this water as it is absorbed by the roots, to the leaves, where it is decomposed.

3. The water absorbed by the roots holds in solution a certain quantity of animal or vegetable matter which contains carbon ; this carbon is carried by the sap into the green parts ; it continues during the night with the oxygen absorbed by them, and in the morning this carbonic acid formed in the leaves, is decomposed by the solar light, as if carbon could not be usefully deposited in the nutritious juices of plants, unless it was formed by the decomposition of carbonic acid.

4. The green parts which are in contact with air or water charged with a small quantity of carbonic acid gas, abstract it, decompose it, and reject the oxygen. If the quantity is more than a twelfth, it acts on the leaf as a poison.

Thus the result of this extensive function, which may be considered as vegetable respiration, is to fix carbon in the plant, whilst the purpose of animal respiration is to diminish the quantity of the same principle. The eudiometric influence of vegetable respiration on the atmosphere, may, perhaps, after these considerations, be estimated with some exactness. Living vegetables vitiate the air, 1. Because all those parts which are not green form carbonic acid with their own carbon and the oxygen of the air. 2d. Because their green parts absorb during the night a small quantity of oxygen gas. The nitrogen exhaled by the flowers is a temporary and very feeble function, not of sufficient consequence to be taken into account. On the other hand vegetables purify the atmosphere by exhaling during the day a marked quantity of oxygen gas, and at the time of vegetation the days are much longer than the nights. This latter effect is more considerable than the former, for the result of vegetation is to increase the quantity of carbon in a vegetable ; now, no molecule of carbon can be fixed there, unless a corresponding quantity of oxygen gas is set free in the air. De Saussure has confirmed this by experiment. He introduced a small branch covered with leaves and still connected with the tree, into a large round bottle of air, the mouth of which he closed. At the end of two or three weeks the air in the bottle was found to contain a greater quantity of free oxygen than before the experiment.

"Thus, experience as well as theory tends to prove, that living vegetables augment daily the quantity of the free oxygen gas of the atmosphere. This is a compensation for the oxygen absorbed in combustion, in animal respiration, and by dead or dying vegetables. The winds unceasingly mix together every part of the atmosphere, so as to make the whole homogeneous, although in certain places one of these functions may be more active than the other. It is by this mechanism that a fixed quantity of oxygen is maintained in the atmosphere, and thus from the humble functions of vegetable life we are led to the exalted contemplation of the universal order of the world."

This extract will give a general notion of the lucid and complete manner in which M. De Caudolle treats his subject; a subject which has excited, we are sorry to say it, very little general interest in this country. There is some truth in what are called popular prejudices, and unquestionably there is a very common disposition to call in question the advantages of the study of botany. If there are any good reasons for this prejudice, they are derived from the mistaken object for which this study is pursued too often. How many after having collected specimens of every species of plants in their neighbourhood, and having pressed them, neatly pasted them on paper, and written beneath them their Latin names, think that they have accomplished all that their opportunities will allow them, and flatter themselves that they are good botanists. But what have they acquired? A knowledge of the shape of the flowers and leaves, and of the number of the pistils and stamens of a few hundred indigenous plants, the first step perhaps to a knowledge of botany, but no more capable of making a botanist in the philosophical acceptation of the term, than would the study of the words in Walker's Dictionary or a Greek Lexicon be sufficient to make an English or a Greek scholar. A similar error is indeed often made in the study of languages; the acquirement of the language itself being considered the ultimate object, and not the stores of knowledge which such an acquisition may put at the student's disposal. This is, as an acute writer has said, making language not the instrument but the end of instruction; as the negro worships his kettle for its own sake and not for its utility. The simile will well apply to the exclusive admirers of the Linnean system. Dr. Lindley has within the last few years energetically endeavoured both as a writer and a lecturer to dispense with an artificial arrangement of plants altogether, and to substitute the so-called natural one; for he had himself felt the unsatisfactory nature of his acquirements even when he was imagined to be an excellent Linnean botanist. Perhaps he has carried his antipathy to his first master too far to be just, for it is very questionable whether any artificial adaptation of the natural system will be equal as an index to that of Linneus; for it must be recollected that however natural the Natural system may be, yet that an artificial division must be appended in order to save the time of the learner in ascertaining the names of those families with which he is unacquainted. But although it might be inconvenient to do away with the Linnean arrangement as an Index, it is not to be regretted, that Mr. Lindley, in his ardour for the promulgation of a more scientific one, has gone to extreme lengths, for he is more likely by such means to excite attention to the true object of artificial classification, and thus to induce more extensive investigations. One of the great benefits of the Natural system is, that its primary divisions are founded on internal structure, and therefore there is a greater probability of its leading the student to inquire into the anatomy and physiology of plants, so as to give a somewhat philosophical character to his investigations, and thus to raise botany in the general opinion to that high rank in the sciences which it undoubtedly should hold. The excellent Introduction to Botany of Dr. Lindley, and the translations of Richard's Elémens de Botanique, will do much to accomplish the same purpose, and those who are disposed to enter more deeply into the study of the structure and functions of plants will find in these volumes of M. De Caudolle, and in those which have preceded them, a fund of matter as valuable and as well arranged as was ever brought together to elucidate any similar subject.

